# The Georgia Press Association

**Published:** 1885-04

**Authors:** 


### THE GEORGIA PRESS ASSOCIATION—THE ANNUAL MEETING IN BAINBRIDGE—A TRIP TO THE EXPOSITION—NOTES BY THE WAY.

On the 17th of March, in company with several members of the Press Association of Georgia, we left Atlanta for the city of Bainbridge, the place fixed for the meeting of the Association. We were joined at Macon by quite a number of editors and their ladies, all intent upon a joyous trip. Our route was via Albany and Thomasville. We arrived in Albany, the Artesian City, about noon. There we were met by a party of citizens and representatives of the press, in company with whom, and under whose conduct, we visited the various artesian wells, in carriages which awaited our coming.

After this pleasant drive, we were feasted at a splendid banquet, given at the Artesian House, in honor of the press.

The people of this beautiful little city are proud of their wells, and they ought to be, for they are proving not only a great comfort and convenience, but are said to be promotive of health to those who use their waters. In conversation with leading physicians of Albany, we learned that the health of that place and vicinity had been greatly improved since the use of artesian waters had become general. Prior to the completion of these wells the only water available for drinking purposes was that which was preserved in cisterns. Now, they have an abundant supply of the very best. The principal well of the city is strongly impregnated with lithia, and nearly all of them contain sulphur.

The boring of wells in various portions of Southern and Southwestern Georgia is certainly a wise and beneficial policy.

From Albany we passed on to Thomasville, the City of Hotels. There we spent the night. Before our arrival we were met on the train by representatives of the press of that city, and informed that, in consequence of the crowded condition of the hotels, we would be entertained at private houses. Among those who met us *en route* was Capt. John Triplett, the accomplished and genial editor of the *Thomasville Times.*

It was our good fortune to have enjoyed the hospitalities of Dr. J. G. Hopkins, to whom, and to his estimable wife, we are indebted for a most delightful sojourn.

Thomasville is now considered the most popular winter resort for consumptives in all the South. There are several large hotels already complete, which are full of Northern visitors, and another will be finished in a few months, having over five hundred rooms. These hotels being required mainly for the accommodation of visitors who seek a Southern climate as a means of relief from pulmonary diseases, some idea may be formed of the great numbers who pay annual visits to this city. Very many have experienced benefits from the genial climate of Southern Georgia.

From this city we went to Bainbridge, where the annual meeting of the Georgia Press Association was held. There we were received with generous hospitality, impressing us most favorably with the noble people of that place. Everything was done which tended to the pleasure and comfort of their visitors. A superb banquet was given the members of the Association, and all vied with each other in making it a pleasant occasion.

At Bainbridge the Press Association received an invitation from the Louisville and Nashville Railroad to visit the Exposition at New Orleans.

Leaving the city of Bainbridge, where we had been so royally entertained, at 9 o'clock on the night of the 18th, we were swiftly and comfortably borne on the excellent line of railroad, arriving early the following morning at Lake DeFuniak, Florida, the Southern Chautauqua. From this place, destined to become one of great note, we had a delightful ride over the Atlantic and Pensacola Railroad to the city of Pensacola. This road has been recently built and is operated by the Louisville and Nashville Company.

We are under obligations to the officers of the Central Railroad, the Savannah, Florida and Western, and the Louisville and Nashville Railroads for special courtesies. All of these officers were unremitting in their attentions, and added much thereby to the pleasures of travel over the splendid roads they represent.

Our trip from Pensacola to Mobile, and to the Crescent city, was marked only by pleasures which completed the unbroken chain of enjoyment that distinguished our entire tour.

Arriving in New Orleans we first devoted ourselves to required rest, after which our attention was naturally and eagerly turned to the great Exposition, in which the civilized world has manifested becoming interest. To write of this immense enterprise in detail would occupy too much space. We must, therefore, refer to it only in general terms. Considering it financially it may not be a success, but when its influence upon the industries of the nation is contemplated it must be admitted that it is accomplishing results of a beneficial nature which are likely to be recognized in this and other countries for many years. We spent several days most pleasantly in viewing the great variety of manufactures, minerals, agricultural productions, inventions, etc., representing the resources, the industries and the genius of the countries that have contributed to this enterprise.

Those who have visited the Exposition express themselves, generally, as well rewarded for their time and expenditures, and we can safely promise that those of our readers who may attend the session of the American Medical Association at New Orleans will be amply repaid.

Georgia, as a State, has no exhibit, but a few enterprising citizens have arranged a very creditable display of minerals and other products of the State. To them the people of Georgia are much indebted. The mortification of proud and patriotic citizens, who find the resources and progress of their great State neglected, is modified only by the work of these public spirited gentlemen.

No State could have made a better exhibit than Georgia if in her capacity as a Commonwealth she had aided the work of revealing her resources and her achievements to the world. This she not only did not do, but in justice to the liberality and patriotism of her people it should be announced, that no appropriation for this purpose could have been made under the restraints of her constitution.

To the Messrs. Pratt, of this city, who had in charge the citizens' exhibit referred to, and to Mr. W. E. Austin, an Atlanta boy, who is in charge of an exhibit for a leading firm at Louisville, we are indebted for highly appreciated courtesies.

The Crescent city possesses many attractions to the stranger, and, independently of the present Exposition, would well reward any one who would make her a visit.

---

### ITEMS.

THERE have been a number of cases of meningitis in the city recently.

LUDDEN & BATES, Savannah, have a very attractive advertisement in this issue of THE JOURNAL.

CORRECTION.—In the article by Dr. H. F. Campbell, in this issue of the JOURNAL, page 76, third paragraph, first line, for *galvano*-faradic current, read faradic current.

INVITATIONS are out to the marriage of Dr. Nathan O. Harris, of this city, and Miss Lula Tucker, of Raleigh, N. C. The marriage will be celebrated in Raleigh on the evening of April 8th.

JUST as we go to press we are called on to announce the death of Dr. J. H. Logan, Professor of Chemistry in the Atlanta Medical College. His death occurred at his home in this city, March 29th. A more extended notice of his death will appear in our next issue.

MR. REID WARE, a prominent young druggist of this city, has just returned from a six months' course at the Philadelphia College of Pharmacy. In a class of three hundred and forty-three he stood among the first in his examination. He has bought an interest in the well-known drug house of L. H. Bradfield, on Whitehall street, and will hereafter be in charge of the prescription department of this house.

